# Supplementary material for: Pain-stimulated ultrasound vocalizations and their impact on pain response in mice
Source: PLoS One. 2025 Jul 9;20(7):e0324730. doi: 10.1371/journal.pone.0324730 (PMC12240302; doi:10.1371/journal.pone.0324730)
Supplement: Attachment — Submitted filename: Response to Reviewers.docx [file pone.0324730.s003.docx]

Dear Dr. Mishra,

Thank you for the opportunity to revise our manuscript (PONE-D-24-35208) titled “Pain-stimulated ultrasound vocalizations and their impact on pain response in mice.” We appreciate the constructive comments and suggestions provided by you and the reviewers, which have greatly contributed to improving our work. Below, we provide a point-by-point response to the comments raised by the academic editor and reviewers, along with a summary of the changes made in the revised manuscript.

Responses to Comments from the Academic Editor:

1. Decibel Range and Sound Stress Duration:

We appreciate the comment regarding sound stress parameters. In the "von Frey test" subsection of the Methods, “We have specified that naïve mice, distinct from those used for recording ultrasound vocalizations, were exposed to sound stress at 80 dB for a duration of 4 hours on day 0 in a soundproof box.” This setup was carefully controlled to deliver consistent sound exposure without additional external stress factors. Sound pressure levels have been reported to influence emotional states in a rat depression model (Yamauchi, *et. al.,* 2025). We believe this clarification ensures transparency regarding the experimental design and adequately addresses the concern. In accordance with the Academic Editor’s comment, we have added the text in **“Methods” (page 7, lines 104 – 107)** as follows:

**“Methods” (page 7, lines 104 – 107)** Naive mice, different from those used for recording cries, were exposed to sound stress at 80 dB for 4 h (day 0) in a soundproof box. This setup was carefully controlled to deliver consistent sound exposure without additional external stress factors. Sound pressure levels have been reported to influence emotional states in a rat depression model [11].

2. Minimizing Suffering, Use of Anesthetic Agents, and the Method of Euthanasia:

We appreciate the comment regarding minimizing suffering, the use of anesthetic agents, and the method of euthanasia. To minimize suffering, euthanasia was performed immediately when humane endpoints were met, following strict animal welfare guidelines. The health and behavior of the animals were monitored daily on weekdays to ensure animal welfare. Additionally, research staff received proper training to ensure the humane handling of animals. These measures underscore our commitment to minimizing animal suffering throughout the study. We use isoflurane as an anesthetic agent in surgical procedures described in the **"Animals" subsection of the Methods (page 6, lines 86)**. The euthanasia procedure was conducted using isoflurane overdose to ensure a painless and humane process. Additionally, thalamic sample collection involved decapitation without anesthesia, performed by appropriately trained researchers. Because anesthesia can induce immediate changes in mRNA expression (Staib-Lasarzik et al., 2014), decapitation without anesthesia was chosen to minimize stress and avoid potential interference with experimental data. Both euthanasia methods were conducted with strict adherence to animal welfare guidelines. In response to the editor's comments, we have revised the manuscript to further clarify these points and emphasize our adherence to ethical standards, explicitly detailing the euthanasia methods used. We have revised the text in **“Methods” (page 6, lines 84 – page 7, lines 95)** as follows:

**“Methods” (page 6, lines 84 – page 7, lines 95)** The duration of each animal experiment was approximately one month each. A total of 177 animals were used, all of which were humanely euthanized. All surgery except for thalamic sample collection was performed under isoflurane anesthesia, with continuous efforts to minimize distress and suffering. When the endpoint was reached, humane euthanasia was carried out without delay. Euthanasia was conducted through isoflurane overdose, a method recognized for being both humane and minimally distressing. Thalamic sample collection was performed by appropriately trained researchers using decapitation without anesthesia. This method was carefully chosen based on evidence that anesthesia can rapidly alter mRNA expression [10]. While minimizing stress and avoiding potential data interference were key considerations, this procedure was conducted strictly under ethical guidelines and with approval from the Institutional Animal Care and Use Committee. All research staff received proper training to ensure the humane handling and care of animals under the guidance of the Institutional Animal Care and Use Committee of Tokyo University of Science and all experiments followed appropriate ethical and technical guidelines.

3. Measurement of Difficulty in Feeding or Drinking and Duration of Pain Monitoring:

We understand the academic editor’s concern regarding how difficulty in feeding or drinking was measured and the potential misinterpretation regarding the duration animals were kept alive after reaching humane endpoints. Humane endpoints were defined as cases where mice exhibited difficulty in feeding or drinking or when they lost 25% or more of their body weight. All animals were closely monitored, and euthanasia was performed immediately if severe pain or distress was observed. Animals meeting humane endpoints were euthanized immediately to prevent any unnecessary distress. We clarify that animals were not kept alive for up to two hours after identifying signs of distress—euthanasia was performed immediately using isoflurane overdose. To prevent any misunderstanding, the manuscript has been revised to provide a clearer explanation of these procedures in **“Methods” (page 6, lines 78 – 83)** as follows:

**“Methods” (page 6, lines 78 – 83)** In this study, humane endpoints were considered, including euthanasia at an appropriate time if the animals experienced intolerable pain or distress. The health and behavior of the animals were monitored daily on weekdays to ensure animal welfare, including checking water bottle usage and food intake. Specifically, humane endpoints were defined as cases where mice exhibit difficulty in feeding or drinking due to stress or pain, or when they lose 25% or more of their body weight. When animals reached the defined endpoints, they were euthanized as quickly as possible. No animals died before meeting the euthanasia criteria.

Responses to Reviewer #1:

Thank you very much for reviewing of our manuscript.

Your comments are highly insightful and enable us to improve the quality of our manuscript. Our responses to the respective comments are described in the following columns. Incorporating these responses, we have revised our manuscript, thus, we would like to resubmit it for publication in *PLOS ONE*.

1. Definition of Nociplastic Pain:

We have carefully reviewed the paper by Kosek et al. and accordance with reviewer’s comment, we judged that we cannot exactly define the pain induced by ultrasound exposure as nociplastic pain. Therefore, we deleted the sentences which were related with nociplastic pain, and revised in **“Introduction” (page 4, lines 39 – 44)** as follows:

**“Introduction” (page 4, lines 39 – 44)** Pain is an important physiological phenomenon that can be a major stressor, sometimes to the point of death, and can occur even in the absence of actual tissue damage [1]. Many reports show that psychological stress enhances pain [2, 3]. In 2016, Smith *et. al.* demonstrated that “bystander” mice housed in the same environment as mice undergoing inflammatory pain exhibited similar hyperalgesia [4]. This emotional transmission is thought to occur because of various information (sight, hearing, smell, etc.) obtained from others; however, their mechanisms are unclear.

2. Experimental Procedures for Thalamus Sample Collection:

Detailed information on the procedures used to collect thalamic samples has been added. The animals were decapitated without anesthesia by appropriately trained researchers to ensure the procedure was performed rapidly and accurately, minimizing stress, and avoiding potential interference of anesthesia with experimental data. The brain regions were then carefully dissected based on anatomical landmarks. The revised text includes a reference to the protocol used in the **“Methods” (page 9, lines 128 – 131)** as follows:

**“Methods” (page 9, lines 128 – 131)** The animals were decapitated without anesthesia by appropriately trained researchers to ensure the procedure was performed rapidly and accurately, minimizing stress, and avoiding potential interference of anesthesia with experimental data. The brain regions were then quickly removed and carefully dissected on ice in a modified manner according to Glowinski and Iversen [15], collecting thalamus from mice a day after sound stress exposure for 4 h (day 1) and subjected to microarray analysis.

3. Model Description and Limitations:

We acknowledge the reviewer's concerns regarding the characterization of this model as one of nociplastic pain. As we did not assess neuronal hyperexcitability in the hypothalamus and/or spinal cord, we recognize that this study alone does not provide sufficient evidence to definitively classify this model as nociplastic pain. In the revised discussion, we emphasize that our primary focus is on pain-related ultrasonic vocalizations and acknowledge that additional experiments—such as assessments of neuronal hyperexcitability and glial activation—are necessary to fully validate the relevance of this model to nociplastic pain.

Furthermore, we agree with the reviewer’s comment that the pain induced by ultrasonic exposure cannot be strictly defined as nociplastic pain, given the observed increase in inflammation-related gene expression in the thalamus of exposed mice. Therefore, our results should be interpreted as revealing a novel pain mechanism induced by ultrasound exposure rather than as definitive proof of nociplastic pain. In accordance with the reviewer's comment, we have revised the manuscript in both **“Introducion”** and **“Discussion”** sections to reflect these points.

**“Introduction” (page 4, lines 54 – 56)** Our study demonstrated that ultrasonic vocalizations emitted by pain stimuli could result in emotional transmission and cause hyperalgesia.

**“Discussion” (page 21, lines 360 – page 22, lines 370)** Pain can be classified into nociceptive, neuropathic, and nociplastic pain. According the International Association for the Study of Pain, nociplastic pain is “pain that arises from altered nociception despite no clear evidence of actual or threatened tissue damage causing the activation of peripheral nociceptors or evidence for disease or lesion of the somatosensory system causing the pain” [30]. We clarified that the pain induced by ultrasonic exposure cannot be strictly defined as nociplastic pain, given the increased inflammation gene expression in the thalamus of exposed mice. However, our results highlight a novel aspect of pain modulation through ultrasonic vocalizations, suggesting that ultrasound exposure may influence pain perception via neuroinflammatory mechanisms. Further investigations, including assessments of neuronal hyperexcitability and glial activation, will be essential to fully elucidate the underlying pathways. Understanding these mechanisms may pave the way for new therapeutic strategies targeting ultrasound-induced pain modulation, ultimately contributing to advances in pain management.

4. Glial Cell Involvement Hypothesis:

We agree with your comment. Following the reviewer’s comment, we have adopted a more cautious approach when discussing the involvement of microglia. The revised discussion now frames this as a hypothesis that warrants further investigation rather than a definitive conclusion in **“Discussion” (page 21, lines 353 – 359)** as follows:

**“Discussion” (page 21, lines 353 – 359)** The hyperalgesia observed in this study was alleviated by SB225002, a CXCR2 antagonist, suggesting that social transfer observed may be associated with neuroinflammatory responses in the thalamus, potentially involving microglial activity. However, further experiments are required to confirm a causal relationship, particularly regarding the balance between M1 and M2 microglia. Nonetheless, the results indicate that CXCR2 antagonists may represent a promising new therapeutic strategy for mitigating hyperalgesia induced by ultrasound exposure.

Responses to Reviewer #2:

We appreciate the reviewer’s acknowledgment of our self-critique regarding the absence of randomized, non-pain-related ultrasonic vocalizations as a control. While we agree that this is a limitation, we believe our current discussion adequately addresses this point and highlights it as an area for future research.

Journal Requirements:

1. Checking that our manuscript conforms to the manuscript style required by PLOS ONE:

We have reviewed and ensured that our manuscript conforms to PLOS ONE’s style requirements, including formatting, file naming, and structure, as outlined in the provided guidelines.

2. Additional information regarding the experiments involving animals

We have provided additional information regarding animals in the **"Animals" subsection of the Methods (page 6, lines 78 – page 7, lines 95)** as follows:

**Methods (page 6, lines 78 – page 7, lines 95)** In this study, humane endpoints were considered, including euthanasia at an appropriate time if the animals experienced intolerable pain or distress. The health and behavior of the animals were monitored daily on weekdays to ensure animal welfare, including checking water bottle usage and food intake. Specifically, humane endpoints were defined as cases where mice exhibit difficulty in feeding or drinking due to stress or pain, or when they lose 25% or more of their body weight. When animals reached the defined endpoints, they were euthanized as quickly as possible. No animals died before meeting the euthanasia criteria.

The duration of each animal experiment was approximately one month each. A total of 177 animals were used, all of which were humanely euthanized. All surgery except for thalamic sample collection was performed under isoflurane anesthesia, with continuous efforts to minimize distress and suffering. When the endpoint was reached, humane euthanasia was carried out without delay. Euthanasia was conducted through isoflurane overdose, a method recognized for being both humane and minimally distressing. Thalamic sample collection was performed by appropriately trained researchers using decapitation without anesthesia. This method was carefully chosen based on evidence that anesthesia can rapidly alter mRNA expression [10]. While minimizing stress and avoiding potential data interference were key considerations, this procedure was conducted strictly under ethical guidelines and with approval from the Institutional Animal Care and Use Committee. All research staff received proper training to ensure the humane handling and care of animals under the guidance of the Institutional Animal Care and Use Committee of Tokyo University of Science and all experiments followed appropriate ethical and technical guidelines.

3. Competing Interests Statement

We confirm that we have included the required statement in the revised manuscript under the "Conflict of Interest" section **(page 21, lines 378 – 380)**, fully complying with PLOS ONE's competing interests policy: “This work was supported by a grant from FUJIMIC Inc. (Tokyo, Japan) and AMED-CREST under Grant Number JP23gm1510008s0102. MS is an employee of FUJIMIC Inc. This does not alter our adherence to PLOS ONE policies on sharing data and materials.”

4. Data Availability Statement:

We have reviewed and ensured that our data availability statement is complete and aligns with PLOS ONE’s open data policy. All data will be made freely accessible upon acceptance, except in cases where public deposition would breach ethical compliance regulations.

5. References:

We have carefully reviewed our reference list to ensure accuracy and completeness. No retracted papers have been cited, and all references have been cross-checked for validity and relevance.

We have uploaded the following files for your consideration:

- Revised manuscript with track changes

- Clean version of the revised manuscript

- Response to reviewers document (this letter)

Thank you for your time and consideration. We look forward to your feedback.

Sincerely,

Satoka Kasai, Ph.D.

Laboratory of Pharmacology and Therapeutics, Department of Pharmacy, Faculty of Pharmaceutical Sciences, Tokyo University of Science

2641 Yamazaki, Noda, Chiba 278-8510, Japan

Tel & Fax: +81-4-7121-3651

E-mail: satoka_kasai@rs.tus.ac.jp
